# Prevalence of porcine respiratory pathogens in slaughterhouses in Shanxi Province, China

**DOI:** 10.1002/vms3.532

**Published:** 2021-05-22

**Authors:** Weidong Yue, Yihui Liu, Yukai Meng, Haili Ma, Junping He

**Affiliations:** ^1^ College of Veterinary Medicine Shanxi Agricultural University Taigu PR China

**Keywords:** *Haemophilus parasuis*, *Mycoplasma hyopneumoniae*, porcine circovirus, porcine reproductive and respiratory syndrome virus, respiratory tract diseases

## Abstract

**Background:**

Porcine respiratory diseases remain the biggest challenge in pig‐based food production and are a public health concern. Despite control measures, persistent outbreaks have been reported worldwide.

**Objective:**

To establish an early detection mechanism for pig farm disease outbreaks based on slaughterhouse risk and environmental assessment.

**Methods:**

We investigated the prevalence and risk factors of porcine respiratory disease‐causing pathogens including Mycoplasma hyopneumoniae (MHP), porcine circovirus type 2 (PCV2), porcine reproductive and respiratory syndrome virus (PRRSV) and Haemophilus parasuis (HPS). Polymerase chain reaction (PCR) was used to analyse the lungs of 491 pigs from 19 slaughterhouses across 11 cities in Shanxi Province, China.

**Results:**

PCR detected MHP, PCV2, PPRSV and HPS in 76.99%, 67.00%, 11.82% and 19.55% of the samples, respectively; 10.12% were negative for all four pathogens. Co‐positivity rates for two and three pathogens were identified. The results confirmed significant correlations between PCV2 and MHP (*p* = .001, *p* < .05), HPS and PCV2 (*p* = .01, *p* < .05) and MHP and PRRSV (*p* = .01, *p* < .05). No significant correlation was observed between HPS and MHP (*p* = .067, *p* > .05). Positive MHP and PCV2 rates were low in areas with high vegetation coverage. The overall pathogen positivity rate was higher in both lower and higher temperature environments.

**Conclusions:**

Interactions among pathogens may increase disease severity. Furthermore, environmental assessment and pathogen surveillance within pig slaughterhouses can be an effective approach for early detection and mitigation of new disease threats before broad dissemination occurs among a herd.

## Graphical abstract

It is a comprehensive epidemiological survey of Mycoplasma hyopneumoniae (MHP), Porcine circovirus type 2 (PCV‐2) and Haemophilus parasuis (HPS) infection in pig in Shanxi of China. 2. The meteorological factors and geographic area might be important variables affecting the transmission of porcine respiratory pathogens. The study suggested that interactions between pathogens might increase the severity of porcine respiratory disease outcomes.

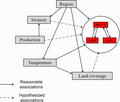

## 1 INTRODUCTION

*Mycoplasma hyopneumoniae* (MHP), porcine circovirus type 2 (PCV2), porcine reproductive and respiratory syndrome virus (PRRSV), and *Haemophilus parasuis* (HPS) have gained importance from an economic perspective in modern pig breeding and rearing worldwide (Charlebois et al., 2014; Martelli et al., 2011; Szeredi et al., 2012). Moreover, co‐infection seems to exert a synergistic effect on disease severity; for example, co‐infection with viruses and/or MHP and opportunistic bacteria results in more severe pneumonia than a single‐infection with any of the pathogens (Thacker et al., 1999). Therefore, it is important to determine the prevalence of MHP, PCV2, HPS and PRRSV, and their co‐infection status. In addition to these pathogen‐specific elements, complex interconnected features, such as environmental and host factors, can influence the spread of some pathogens (Niederwerder et al., 2016; Ostanello et al., 2007). For instance, low temperatures or other climatic factors can affect interactions between the host, pathogen and the environment, thus increasing the probability of exposure to and infection by the pathogen (Jean‐Baptist et al., 2009).

China produces around 49% of the global pig population, and the Shanxi Province in northern China is among the largest contributors to China's swine industry (Beltran‐Alcrudo et al., 2019). In some areas, the slaughterhouses are an important checkpoint for assessing the health status of pigs and they are an important source of epidemiological data in British pig health schemes (Sanchez‐Vazquez et al., 2011). Understanding and building infection risk‐based awareness in slaughterhouses are essential to prevent and monitor disease outbreaks in pig farms. Therefore, in this study, we aimed to assess the positivity rates of PCV2, MHP, PRRSV and HPS in large‐scale pig slaughterhouses in Shanxi Province, along with other potential factors (such as meteorological and geographic factors) that influence infection rates.

## 2 MATERIALS AND METHODS

### 2.1 Sample collection

**FIGURE 1:**
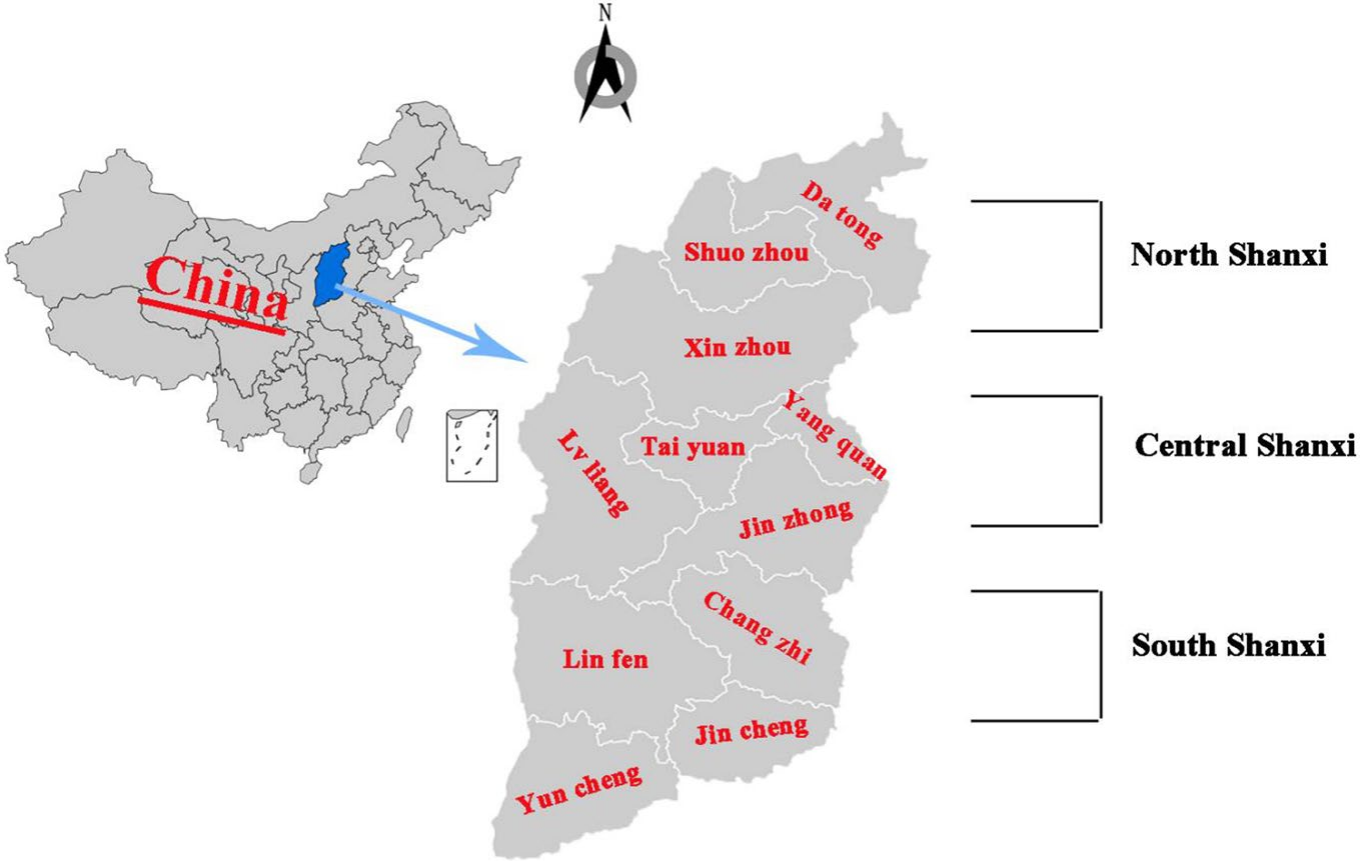
Pig sampling locations in Shanxi Province, China. The blue area indicates the sampling locations and sample collection cities

Lung tissue samples (*n* = 491; 5–7‐month‐old pigs) were collected from 19 pig slaughterhouses were major large‐scale slaughterhouses in Shanxi Province, which were distributed in all 11 cities and cover the pig farms in the 50 counties of Shanxi Province, accounting for about 14% of the total slaughterhouses. The samples were collected between March and June in 2019. The daily slaughter capacity of the slaughterhouse was about 1000–2000. Sampling coverage area map in Shanxi Province (Figure 1). Lung tissues were randomly selected from the slaughter line of pigs, put into a sterile bag containing tissue protection solution, placed in an icebox, transported back to the laboratory for sterile separation and storage in a cryopreservation tube, and stored at −80°C for long‐term preservation.

### 2.2 DNA isolation and polymerase chain reaction (PCR)

RNA and DNA were extracted from the collected tissue samples using the RNAprep Pure Tissue Kit and the TIANamp Genomic DNA Kit (TIANGEN) according to the manufacturer's instructions. MHP, PCV2 with HPS and PRRSV were detected using nested PCR (N‐PCR), PCR and reverse transcription (RT)‐PCR. The primer sequences used in this study are listed in Table 1. The PCR products were analysed using gel electrophoresis on a 2% agarose gel in Tris‐acetic‐acid‐EDTA buffer and stained with GelRed™ (Biosharp, Hefei, China). The bands were visualised under ultraviolet illumination.

**TABLE 1:** Primer sequences used for the survey of pathogens of porcine respiratory diseases

Pathogen	Assay	Sequence	Product size	References
MHP	*N*‐PCR	P1:5ʹ‐TTAGTGTCTCCCGTTATG−3ʹ	621bp	This study
		P2:5ʹ‐GAAATCCGTATTCTCCTC−3ʹ		
		P3:5ʹ‐TTACAGCGGGAAGACC−3ʹ	427 bp	
		P4:5ʹ‐CGGCGAGAAACTGGATA−3ʹ		
PCV2	PCR	P1:5ʹ‐TTTAGG GTTTAAGTGGGG GGTC−3ʹ	470 bp	Zhou et al., 2006
		P2:5ʹ‐CCGGATCCATGACGTACCCAAGGAGGCG−3ʹ		
HPS	PCR	P1:5ʹ‐GTGATGAGGAAGGGTGGTGT−3ʹ	821 bp	Angen et al., 1998
		P2:5ʹ‐GGCTTCGTCACCCTCTGTA−3ʹ		
PRRSV	RT‐PCR	P1:5ʹ‐GGCGACCGTTTTAGCCTGTCTT−3ʹ	735 bp	Liang et al., 2018
		P2:5ʹ‐ATCATTATTGGCGTGTAGGTG−3ʹ		

Abbreviations: HPS, *Haemophilus parasuis*; MHP, *Mycoplasma hyopneumoniae*; *n*‐PCR, nested PCR; PCR, polymerase chain reaction; PCV2, porcine circovirus type 2; PRRSV, porcine reproductive and respiratory syndrome virus; RT‐PCR, reverse transcriptase‐PCR.

### 2.3 N‐PCR

In the first step, the N‐PCR reaction mixture contained 1.5 μL extracted DNA, 2 μL primer pairs (10 μM), 10 μL PCR Master Mix (Vazyme) and 6.5 μL RNase‐free water. In the second step, the N‐PCR reaction mixture contained 0.5 μL of the product DNA obtained in the first step, 2 μL primer pairs (10 μM), 10 μL PCR Master Mix and 7.5 μL RNase‐free water. PCR amplification conditions were as follows: pre‐denaturation at 95°C for 5 min; 34 cycles of denaturation at 95°C for 30 s, annealing at 42°C for 30 s and extension at 72°C for 1 min; and a final extension at 72°C for 8 min.

### 2.4 PCR

The PCR mixture contained 1.5 μL extracted DNA, 1 μL primer pairs (10 μM), 10 μL PCR Master Mix and 6.5 μL RNase‐free water. PCR amplification conditions for PCV2 DNA detection were as follows: pre‐denaturation at 94°C for 4 min; 30 cycles of denaturation at 94°C for 1 min, annealing at 55°C for 1 min and extension at 72°C for 1 min; and a final extension at 72°C for 7 min. PCR amplification conditions for HPS DNA detection were as follows: pre‐denaturation at 94°C for 5 min; 35 cycles of denaturation at 94°C for 45 s, annealing at 56°C for 45 s and extension at 72°C for 1 min; and a final extension at 72°C for 10 min.

### 2.5 RT‐PCR

RT‐PCR was performed using a HiScript II One Step RT‐PCR Kit (Dye Plus; Vazyme). The amplification conditions were as follows: pre‐denaturation at 95°C for 5 min; 35 cycles of denaturation at 95°C for 30 s, annealing at 60°C for 30 s and extension at 72°C for 30 s; and a final extension at 72°C for 10 min.

### 2.6 Analysis of geographical data

Land cover data were included in the analysis and obtained from the Data Centre for Resources and Environmental Sciences, Chinese Academy of Sciences (http://www.resdc.cn). The land cover data were all inputted into a raster map through ArcGIS 10.4 software. Vegetation coverage was represented by optical density analysis using ImageJ. The SPSS software independence test was used to analyse the correlation between the positivity rates of MHP and PCV2 and geographical area.

### 2.7 Analysis of temperature data

The 2019 temperature data for the whole Province were collected, including the average, minimum and maximum daily temperatures for each season. Multivariate correlation analysis was performed to compare the data for temperature and positivity rates.

### 2.8 Statistical analyses

Pathogen‐specific PCR results were analysed as qualitative (positive/negative) data. Statistical significance was determined using one‐way analysis of variance. Differences in the distribution of pathogens among samples were analysed using chi‐squared tests. To determine the correlations among PCV2, MHP, HPS and PRRSV, the Pearson correlation method was applied to the data. SPSS software, Image J (National Institutes of Health), and GraphPad Prism 5.0 (GraphPad) were used for correlation and statistical analysis. Significance was set at the 0.01 level (bilateral) (*p* = .001, *p* < .05).

## 3 RESULTS

### 3.1 Pathogen positivity rates

**FIGURE 2:**
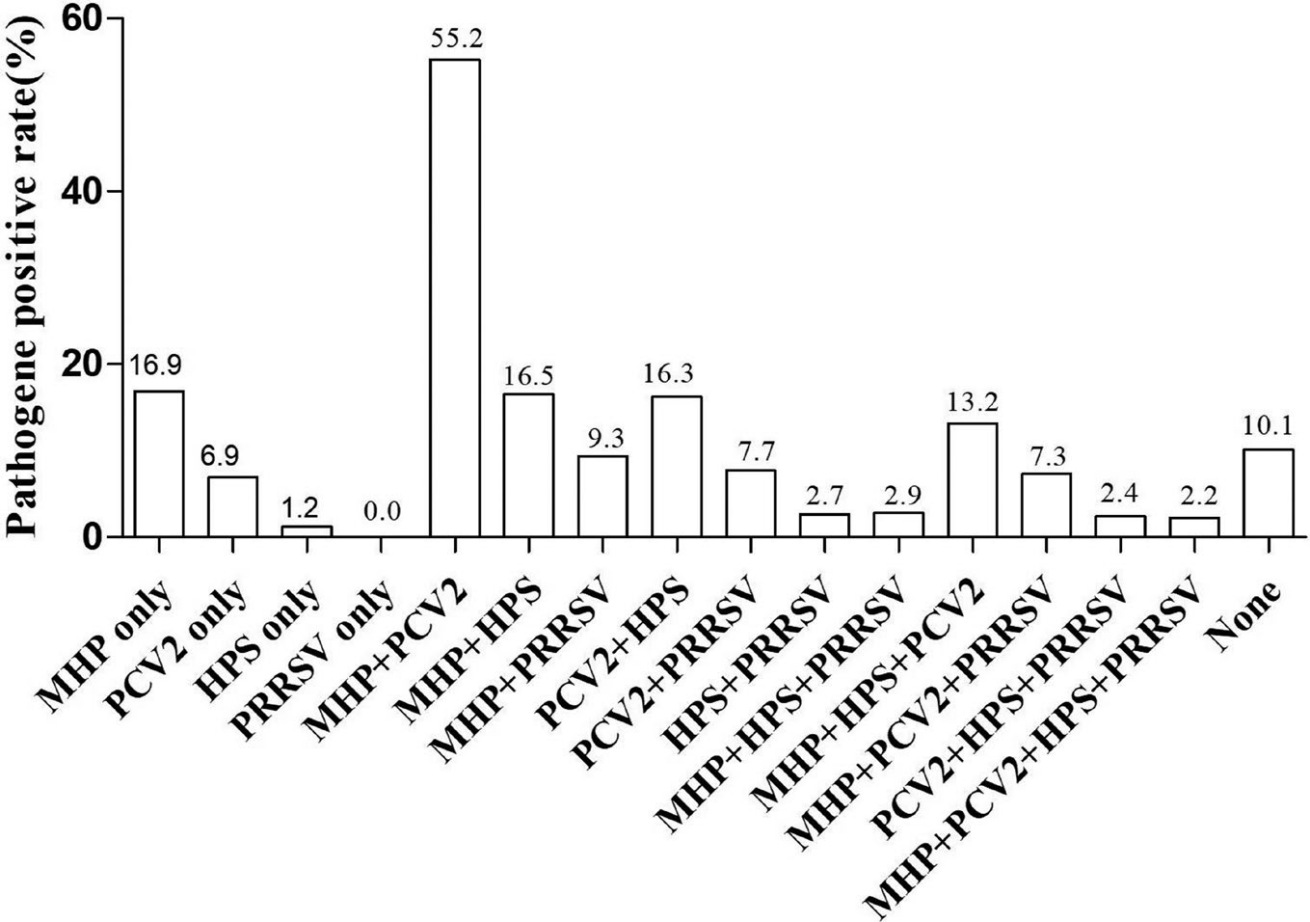
2Proportions of major pathogens and major pathogen combinations in PCR‐positive porcine samples. MHP, *Mycoplasma hyopneumoniae*; PCV2, porcine circovirus type 2; HPS, *Haemophilus parasuis*; PRRSV, porcine reproductive and respiratory syndrome virus

The positivity rates of PCV2, MHP, PRRSV and HPS in lung tissue samples (*n* = 491) were 67.00, 76.99, 13.44 and 19.55%, respectively (Table 2). The major pathogen combinations identified from the PCR data are summarised in Figure 2, Table 3, and Table 4. In general, the detection rate for all pathogens was the lowest in the Jinzhong area of central Shanxi. Co‐infections with multiple pathogens were more common than single‐pathogen infections. The results confirmed significant correlations between PCV2 and MHP (*p* = .001, *p* < .05), HPS and PCV2 (*p* = .01, *p* < .05), and MHP and PRRSV (*p* = .01, *p* < .05). No correlation was observed between HPS and MHP (*p* = .067, *p* > .05).

**TABLE 2:** Prevalence of MHP, PCV2, HPS and PRRSV in Shanxi province

Region	City	Pathogen positivity rate (*n*) %			
		MHP	PCV2	HPS	PRRSV
Northern Shanxi	Datong	(24/27) 88.89	(14/27) 51.85	(0/27) 0	(0/27) 0
	Shuozhou	(41/52) 78.45	(37/52) 71.15	(17/52) 32.69	(13/52) 25.00
	Xinzhou	(46/52) 88.46	(42/52) 80.77	(8/52)15.38	(0/52) 0.00
Central Shanxi	Taiyuan	(11/22) 50.00	(9/22) 40.91	(0/22) 0	(0/22) 0.00
	Yangquan	(13/28) 46.43	(6/28) 21.43	(028) 0	(5/28) 17.86
	Lüliang	(35/54) 64.82	(32/54) 59.26	(2/54) 3.70	(8/54) 14.81
	Jinzhong	(37/49) 75.51	(35/49) 71.43	(17/49) 34.69	(0/49) 0.00
Southern Shanxi	Linfen	(34/50) 68.00	(33/50) 66.00	(7/50) 14.00	(2/50) 4.00
	Changzhi	(48/54) 88.89	(41/54) 75.93	(22/54) 40.74	(0/54) 0.00
	Yuncheng	(44/51) 86.27	(38/51) 74.51	(10/51) 19.61	(4/51) 7.84
	Jincheng	(45/52) 86.54	(42/52) 80.77	(13/52) 25.00	(22/52) 42.31
	Total	(378/491) 76.99	(329/491) 67.00	(96/491) 19.55	(58/491) 11.82

Abbreviations: HPS, *Haemophilus parasuis*; MHP, *Mycoplasma hyopneumoniae*; PCV2, porcine circovirus type 2; PRRSV, porcine reproductive and respiratory syndrome virus.

**TABLE 3:** Prevalence of MHP, PCV2 and HPS co‐positivity in Shanxi province

City	Pathogen positivity rate (*n*) %			
	MHP + PCV2	PCV2 + HPS	HPS + MHP	HPS + MHP + PCV2
Datong	(13/27) 48.15	(0/27) 0.00	(0/27) 0.00	(0/27) 0.00
Shuozhou	(31/52) 59.62	(15/52) 28.85	(16/52) 30.77	(15/52) 28.85
Xinzhou	(39/52) 75.00	(6/52) 11.54	(8/52) 15.38	(6/52) 11.54
Taiyuan	(0/22) 0	(0/22) 0.00	(0/22) 0.00	(0/22) 0.00
Yangquan	(2/28) 7.14	(0/28) 0.00	(0/28) 0.00	(0/28) 0.00
Lüliang	(23/54) 42.59	(1/54) 1.85	(2/54) 3.70	(0/54) 0.00
Jinzhong	(25/49) 51.02	(12/49) 24.49	(14/49) 28.57	(9/49) 18.37
Linfen	(22/50) 44.00	(5/50) 10.00	(2/50) 4.00	(2/50) 4.00
Changzhi	(34/54) 62.96	(21/54) 38.89	(19/54) 35.19	(17/54) 31.48
Yuncheng	(35/51) 68.63	(8/51) 15.69	(8/51) 15.69	(8/51) 15.69
Jincheng	(41/52) 78.85	(12/52) 23.08	(12/52) 23.08	(9/52) 17.31
Total	(270/491) 54.99	(80/491) 16.30	(81/491) 16.50	(66/491) 13.44

Abbreviations: HPS, *Haemophilus parasuis;*MHP, *Mycoplasma hyopneumoniae*; PCV2, porcine circovirus type 2.

**TABLE 4:** Prevalence of MHP, PCV2, HPS and PRRSV co‐positivity in Shanxi province

City	Pathogen positivity rate (*n*) %					
	MHP + PRRSV	PCV2 + PRRSV	HPS + PRRSV	PRRSV + MHP + PCV2	PRRSV + HPS + PCV2	PRRSV + MHP + PCV2 + HPS
Datong	(0/27) 0.00	(0/27) 0.00	(0/27) 0.00	(0/27) 0.00	(0/27) 0.00	(0/27) 0.00
Shuozhou	(11/52) 21.15	(11/52) 21.15	(4/52) 7.70	(7/52) 13.46	(4/52) 7.69	(4/52) 7.69
Xinzhou	(0/52) 0.00	(0/52) 0.00	(0/52) 0.00	(0/52) 0.00	(0/52) 0.00	(0/52) 0.00
Taiyuan	(0/22) 0.00	(0/22) 0.00	(0/22) 0.00	(0/22) 0.00	(0/22) 0.00	(0/22) 0.00
Yangquan	(3/28) 10.71	(4/28) 12.29	(0/28) 0.00	(2/28) 7.14	(0/28) 0.00	(0/28) 0.00
Lüliang	(8/54) 14.81	(4/54) 7.41	(0/54) 0.00	(7/54) 12.96	(1/54) 1.85	(0/54) 0.00
Jinzhong	(0/49) 0.00	(0/49) 0.00	(0/49) 0.00	(0/49) 0.00	(0/49) 0.00	(0/49) 0.00
Linfen	(2/50) 4.00	(2/50) 4.00	(0/50) 0.00	(1/50) 2.00	(0/50) 0.00	(0/50) 0.00
Changzhi	(0/54) 0.00	(0/54) 0.00	(0/54) 0.00	(0/54) 0.00	(0/54) 0.00	(0/54) 0.00
Yuncheng	(3/51) 5.88	(3/51) 5.88	(2/51) 3.92	(2/51) 3.92	(2/51) 3.92	(1/51) 1.96
Jincheng	(18/52) 34.62	(12/52) 23.08	(7/52) 13.46	(17/52) 32.69	(7/52) 13.46	(8/52) 13.38
Total	(28/491) 5.70	(36/491) 7.33	(13/491) 2.65	(36/491) 7.33	(14/491) 2.85	(13/491) 2.65

Abbreviations: HPS, *Haemophilus parasuis*; MHP, *Mycoplasma hyopneumoniae*; PCV2, porcine circovirus type 2; PRRSV, porcine reproductive and respiratory syndrome virus.

### 3.2 Correlation between land cover and pathogen positivity

**FIGURE 3:**
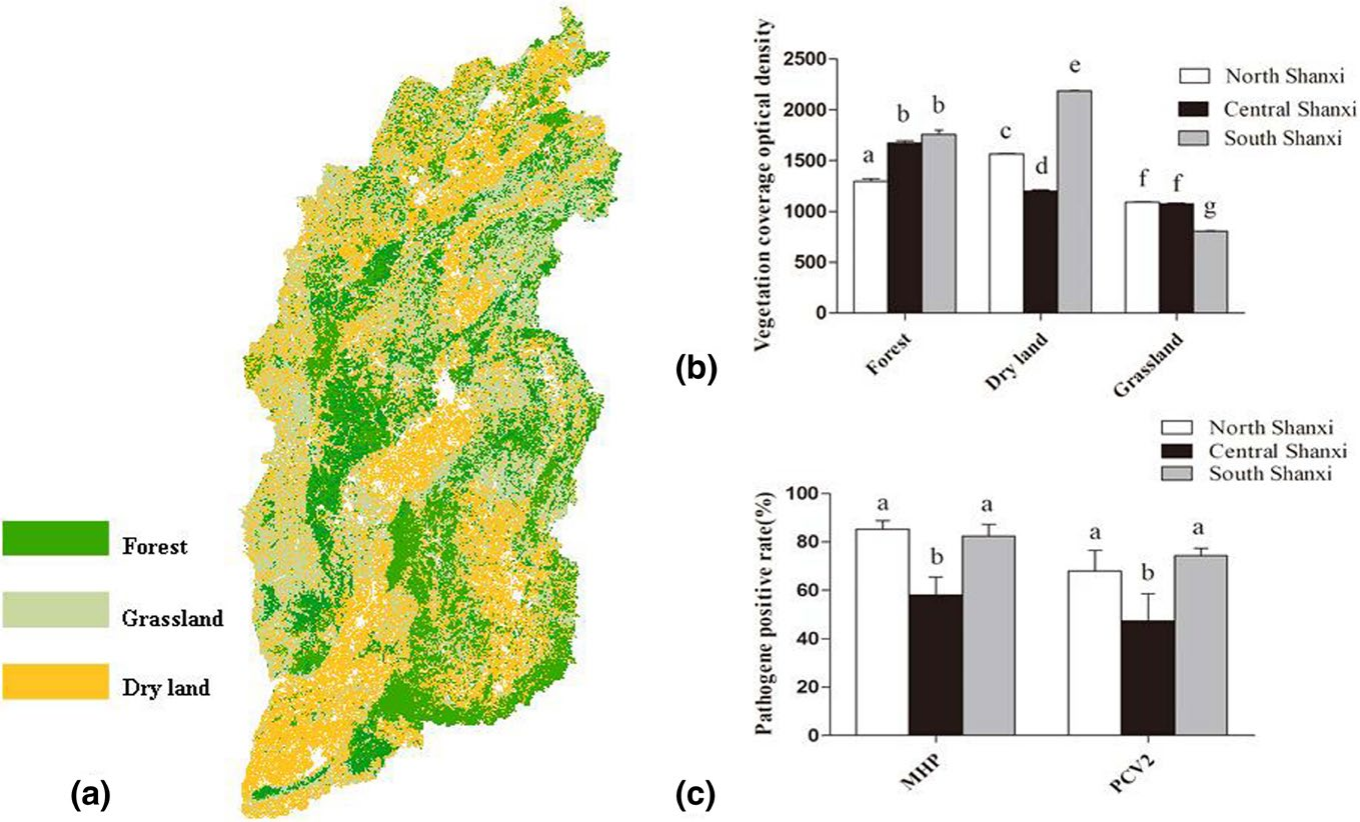
Correlation between land cover and pathogen positivity rate. (a) ArcGIS 10.4 software was used to import all land cover data into raster maps. (b) Vegetation cover distribution using ImageJ optical density analysis (*p* < .05). (c) Positivity rates for *Mycoplasma hyopneumoniae* and porcine circovirus type 2 in the Shanxi province (*p* < .05)

ArcGIS 10.4 (http://www.resdc.cn) was used to input land cover data for Shanxi Province into a raster map. No correlation was found between the HPS positivity rate and the geographical area. The PRRSV positivity rate was the highest in southern Shanxi Province. Data indicated that the forest cover were mainly distributed in central and southern Shanxi; grassland cover was mainly distributed in northern and southern Shanxi, and the arid cover was mainly distributed in southern Shanxi (Figure 2b). As shown in Figure 2c, the positivity rates of MHP and PCV2 were high in northern and southern Shanxi but low in central Shanxi (*p* < .05). The regional sampling test results showed a correlation between MHP (*χ* ^2^ = 29.035, *df* = 2, *p* < .05) and PCV2 (*χ* ^2^ = 194.290, *df* = 3, *p* < .05) positivity rates (Figure 3)

### 3.3 Correlation between environmental temperature and pathogen positivity

MHP, PCV2, PRRSV and HPS positivity rates (Table 2) and environmental temperatures (Table 5) showed a significant correlation (*p* = .001, *p* < .05). The pathogen positivity rate was found to increase in both lower and higher temperature environments.

**TABLE 5:** The temperature in Shanxi province

Region	Average temperature (℃)			
	Spring	Summer	Autumn	Winter
Northern Shanxi	−2.9	17.1	20.4	1.6
Central Shanxi	0.7	19.3	22.7	4.9
Southern Shanxi	3.7	20.8	24.9	6.9

## 4 DISCUSSION

We investigated the prevalence and risk factors of porcine respiratory pathogens in slaughterhouses in Shanxi Province, China. Our results indicated that the positivity rates of MHP and PCV2 were the lowest in the regions with the highest percentage of grassland and forest area, whereas they were the highest in the regions with the highest percentage of dry land (*p* < .05). The PRRSV positivity rate was the highest in southern Shanxi, which has a high forest coverage. This result was consistent with that reported by Arruda (Arruda et al., 2017). Southern Shanxi is among the most swine‐dense regions in the country and it has a high cultivated area. However, from December to March, the cultivated land in this region remains barren and, together with the high temperatures and strong winds, it provides an environment conducive to the spread of pathogens. Therefore, the area can be exposed to large amounts of airborne virus particles released by potentially infected herds (Velasova et al., 2012). The low forest cover in northern Shanxi can lead to the spread of pathogens through environmental media, droplets, dust and vehicles. By contrast, central Shanxi, mainly located in the basin area, has lush vegetation and low winds, making it an unfavourable environment for pathogen transmission. The most important insight of this study is that land cover appears to serve as a protective factor against pathogen transmission.

Previous studies have indicated that in winter and spring, when temperatures change and decrease quickly, pathogens causing respiratory diseases spread faster and infection rates rise significantly (Palzer et al., 2008). Therefore, temperature was considered as the main contributor to the seasonal variation of MHP and HPS infection rates (Browne et al., 2017; Segalés et al., 2012; Wang et al., 2017). Other studies have also shown that lower temperatures facilitate the formation of PRRSV aerosols, which further promotes virus transmission (Joseph et al., 2007). These results concur with those of the present study, in which a higher frequency of respiratory problems and transmission of respiratory pathogens were found at lower environmental temperatures. Interestingly, in the present study, the positivity rates of MHP, PCV2, PPRSV and HPS were higher in southern Shanxi, where the highest temperatures were recorded, than those in central Shanxi (*p* < .05). Thus, higher temperatures may also increased the risk of pathogen transmission.

Numerous risk factors play important roles and several pathogens are involved in the pathogenesis of respiratory diseases (Fablet et al., 2012). Co‐infection with bacteria and viruses has a synergistic effect on the severity of respiratory diseases (Palzer et al., 2008). In this study, MHP, PCV2, PRRSV and HPS were all found to potentially increase the risk of pneumonia. Previous investigations have revealed a significant positive correlation between MHP and PCV2 in healthy pigs (Hansen et al., 2010; Megan et al., 2016), and MHP infection often occurs concomitantly with PCV2 or HPS infections (Garcia‐Morante et al., 2017; Wellenberg et al., 2010). Here, positive correlations were observed between MHP and PCV2 (*p* < .05) and between MHP and HPS (*p* < .05) infections. The MHP positivity rate was the highest among all combinations and single pathogens. Furthermore, MHP and PCV2 showed the highest positivity rate among the pathogen combinations, and PRRSV infection was also often accompanied by MHP infection. Thus, the consistent presence of MHP, in combination and individually, and the lower prevalence of other viruses and bacteria in all the samples potentially indicate the importance of MHP in porcine respiratory diseases.

## 5 CONCLUSIONS

The advancement of modern agricultural production has resulted in improved pig production; however, meteorological and geographical factors may influence infectious disease transmission. Furthermore, interactions between pathogens have also been indicated to increase disease severity. Therefore, environmental assessment and pathogen surveillance within pig slaughterhouses can be an effective approach for the early detection of new disease threats and their mitigation before broad dissemination occurs among a herd.

## CONFLICT OF INTEREST

The authors declare no conflict of interest.

## AUTHOR CONTRIBUTIONS

Conceptualisation: Junping He, Data curation: Haili Ma, Formal analysis: Weidong Yue, Funding acquisition: Haili Ma, Investigation: Yihui Liu, Methodology: Weidong Yue, Project administration: Junping He, Resources: Xinrong Zhang, Software: Yukai Meng, Supervision: Junping He, Visualisation: Yihui Liu, Writing—original draft: Weidong Yue, Writing—review and editing: Haili Ma.

### PEER REVIEW

The peer review history for this article is available at https://publons.com/publon/10.1002/vms3.532.
